# SARS-CoV-2-Indigenous Microbiota Nexus: Does Gut Microbiota Contribute to Inflammation and Disease Severity in COVID-19?

**DOI:** 10.3389/fcimb.2021.590874

**Published:** 2021-03-11

**Authors:** Indranil Chattopadhyay, Esaki M. Shankar

**Affiliations:** Department of Life Sciences, Central University of Tamil Nadu, Thiruvarur, India

**Keywords:** ACE2, COVID-19, Dysbiosis, Microbiota (microorganism), SARS-CoV-2 (2019-nCoV), Inflammation

## Abstract

Gut microbiome alterations may play a paramount role in determining the clinical outcome of clinical COVID-19 with underlying comorbid conditions like T2D, cardiovascular disorders, obesity, etc. Research is warranted to manipulate the profile of gut microbiota in COVID-19 by employing combinatorial approaches such as the use of prebiotics, probiotics and symbiotics. Prediction of gut microbiome alterations in SARS-CoV-2 infection may likely permit the development of effective therapeutic strategies. Novel and targeted interventions by manipulating gut microbiota indeed represent a promising therapeutic approach against COVID-19 immunopathogenesis and associated co-morbidities. The impact of SARS-CoV-2 on host innate immune responses associated with gut microbiome profiling is likely to contribute to the development of key strategies for application and has seldom been attempted, especially in the context of symptomatic as well as asymptomatic COVID-19 disease.

## Introduction

Severe acute respiratory syndrome coronavirus 2 (SARS-CoV-2) is responsible for the development coronavirus disease 2019 (COVID-19) globally, described initially from a wet market in Wuhan, China, back in September 2019 (Chan et al., 2020). The World Health Organization (WHO) has reported 114,751,575 confirmed global cases and 2,549,260 deaths as of 3rd March 2021 due to COVID-19 (He Y et al., 2020; Shankar et al., 2021). The high rates of morbidity and mortality in COVID-19 results from the onset of a severe acute respiratory distress syndrome (ARDS) and systemic inflammatory response syndrome (SIRS), which afflicts the pulmonary compartment initially eventually leading to multi-organ failure (MOF) (Chen et al., 2020). The predominant involvement of the respiratory system in COVID-19 pathogenesis mainly stems from the mode of entry of the virus into the host, i.e., respiratory tract, and also owing to the high expression of angiotensin-converting enzyme 2 (ACE2), the classical receptor to which the viral spike protein ligand can engage with, on the respiratory and the gastrointestinal epithelia. Reports also suggest that SARS-CoV-2 inhibits the absorption of nutrients in the GI tract that drive the onset of gastroenteritis in a sizeable number of affected individuals (Zhou et al., 2020).

The human gut microbiota represents a highly complex and dynamic microbial community that plays a critical role in protecting the host from pathogenic microbial invaders (Vemuri et al., 2018). An extensive and integrated network of gut microbiota works in concert with the host to promote health, and any event that disrupts the homeostasis likely entails disease development (Vemuri et al., 2020). It is widely believed that the diversity of gut microbiota directly impacts the overall health of the host. It is also likely that alterations in gut microbiome could determine the natural history and clinical outcome of COVID-19, together with the described underlying co-morbid conditions like type 2 diabetes (T2D), cardiovascular disorders, and obesity warranting manipulation of the gut microbiota by employing several combinatorial approaches.

## Overview of ACE2-Microbiota Nexus in Host Immunity 

It has been shown that ACE2 expression reportedly alters the lung and gut microbiomes during certain underlying conditions involving the cardiac and pulmonary compartments (Cole-Jeffrey et al., 2015). It has also been known that ACE2 is involved in regulating inflammation and maintaining a healthy microbial community in the host (Hashimoto et al., 2012). Gut microbiome is involved in the regulation of genes involved in immune responses and metabolism in the host (Li et al., 2019). Furthermore, ACE2 has been shown to regulate intestinal metabolism of tryptophan, which drives the release of antimicrobial peptides to maintain and sustain the composition of gut microbiota. It has become clear that down-regulation of ACE2 reduces the intestinal absorption of tryptophan that lowers the secretion of antimicrobial peptides entailing gut dysbiosis (He Y. et al., 2020). Bacterial species such as *Bacteroides dorei* appear to have a significant role in regulating host immune responses by suppressing the expression of colonic ACE2 (Yoshida et al., 2018) supported by the finding that critically ill COVID-19 patients develop gastrointestinal symptoms (Du et al., 2020). Hence, gut microbiota seemingly plays a determining role in SARS-CoV-2 infection through the gut-lung axis (Marsland et al., 2015). Evidence suggests that influenza virus-driven lung injury could be enhanced by alteration in gut microbiota that in turn is believed to be associated with a significant reduction in host antiviral surveillance (Ichinohe et al., 2011). Interestingly, over-expression of fecal calprotectin is suggestive of the role of gut inflammation as a critical baseline finding in clinical COVID-19 (Zuo et al., 2020). Hence, it is imperative to address the role of indigenous microbiota as a key target in the development of anti-SARS-CoV-2 therapeutics and strategies.

## Gut-Lung Microbiome Axis: Lessons Learnt from Classical Respiratory Viral Infections

The gut microbiome is reportedly involved in digestion and protection against pathogens in the host (Hall et al., 2017), and often encompasses phyla Actinobacteria, Firmicutes, Proteobacteria, and Bacteroidetes. The healthy human colon has a greater abundance of bacterial families such as Bacteroidaceae, Prevotellaceae, Rikenellaceae, Lachnospiraceae, and Ruminococcaceae. Evidence suggests that the gut has a preponderance of Bacteroidetes and Firmicutes whereas the pulmonary compartment harbors a considerable population of Bacteroidetes, Firmicutes, and Proteobacteria (Zhang et al., 2020). Gut microbiota appears to have a serious impact on lung infection mediated *via* the gut-lung axis (Dumas et al., 2018). Gut microbiota regulates the optimal functioning of the innate and adaptive immune systems, and antimicrobial peptides and secondary metabolites derived from intestinal commensals are involved in cellular homeostasis (Negi et al., 2019a). Microbe-associated molecular patterns (MAMPs) as well as pathogen-associated molecular patterns (PAMPs) are recognized by toll-like receptors (TLRs) of host cells that drive the regulation of pro- and anti-inflammatory signals (Negi et al., 2019b), and hence a depletion in gut microbial diversity could inflict significant damage on host health (Mosca et al., 2016). Gut commensals, bacteroides, lactobacillus, and bifidobacteria reportedly release a plethora of short-chain fatty acids (SCFA) such as butyrate, acetate and propionate, which bind with dendritic cells (DCs) and macrophages that drive immunomodulation (Jia et al., 2018). Recent findings suggest that gut microbiota could play a significant role in the induction of ARDS (Dickson, 2016) setting clues to advance our understanding of the likely possibility of the same in determining the onset of SARS-CoV-2-mediated tissue damage originating from hypercytokinemia (Girija et al., 2020) and SIRS (Dhar and Mohanty, 2020). Respiratory viral infections such as influenza virus and respiratory syncytial virus (RSV) reflect a significant impact of alteration in gut microbiome with disease severity and prognosis (Deriu et al., 2016). Influenza virus infection induces alterations of gut microbiome through a type I interferon-dependent mechanism (Abt et al., 2012). It appears that the normal gut microbiota elicits the activation and assembly of inflammasome and T-cell responses by inducing the migration of DCs and macrophages in influenza virus infection (Steed et al., 2017). Microbe-derived secondary metabolites such as SCFAs, produced commonly by bacteroidetes and/or clostridia enhanced the protection attributes against influenza virus infection largely by inducing CD8+ T-cell functions and type I IFN signaling in macrophages (Abt et al., 2012). Hence, gut microbiome plays a significant role in regulating immune responses in systematic and distant mucosal sites, including the lungs (Gu et al., 2020).

**Figure 1:**
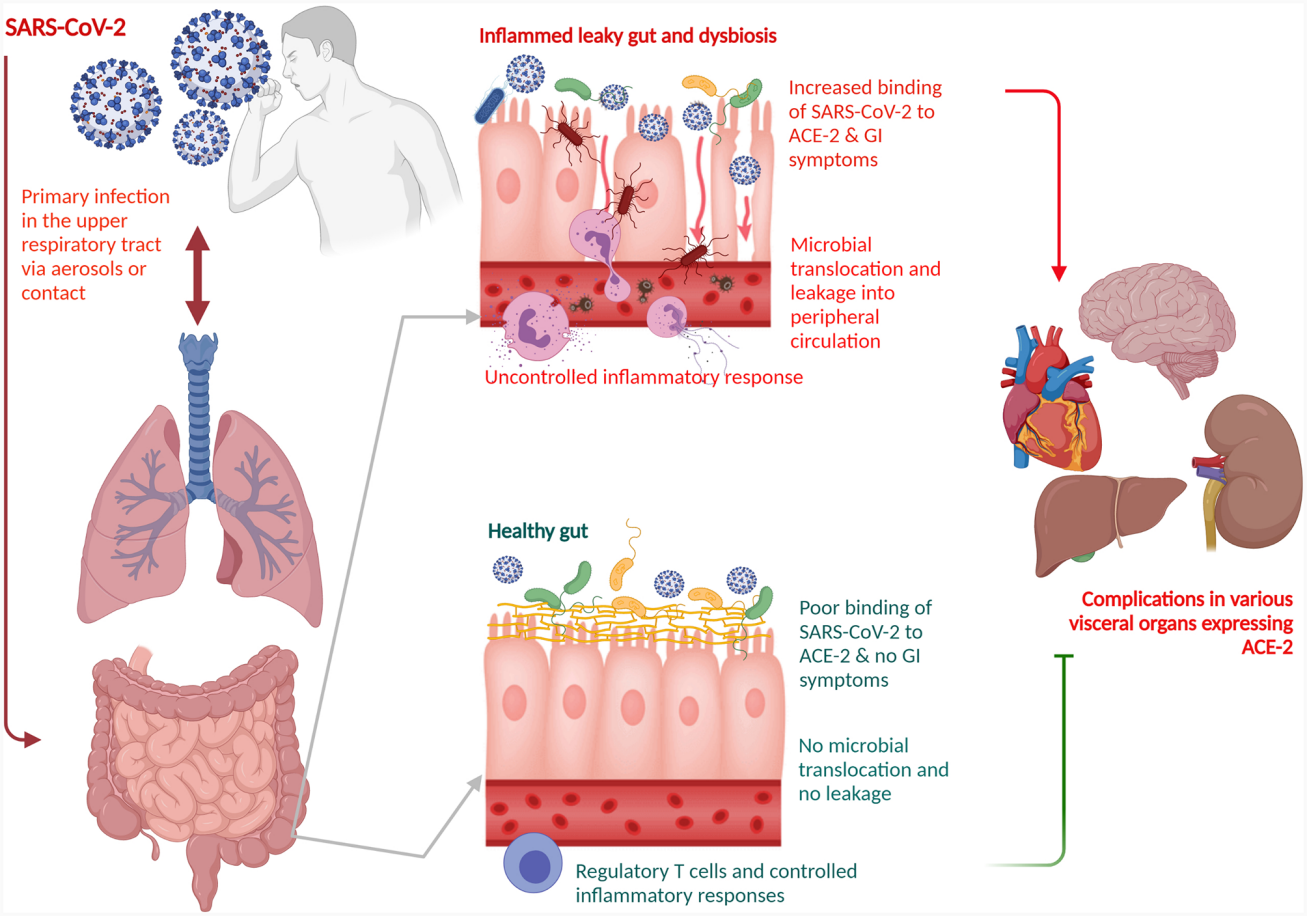
Schematic representation of a role of gut-lung axis with dysbiosis of the gut microbiota for COVID-19 disease management. The gut-lung axis is bidirectional which means metabolites that are derived from gut bacteria effect on the lung through blood whereas inflammation of the lung modifies the level of gut microbiota. SARS-CoV-2 primarily affects the lung. Dysbiosis of gut microbiota breaks the integrity of gut barrier which induces the translocation of SARS-CoV-2 from the lung into the gut through the circulatory and lymphatic systems. The virus binds with the ACE2 on the enterocytes. Inflammation in the gut induces leaky gut that drives the development of sepsis in COVID-19 patients through translocation of bacterial antigens and toxins into the systemic circulation. Dysbiosis of gut microbiota also induces pathogenesis in the lung of respiratory distress syndrome. SARS-CoV-2, severe acute respiratory syndrome coronavirus 2; ACE2, angiotensin-converting enzyme 2.

The gut-lung axis is bidirectional which means metabolites that are derived from gut bacteria effect on the lung through blood whereas inflammation of the lung modifies the level of gut microbiota. Dysbiosis of gut microbiota induces lung dysfunction *via* alteration of immune responses of neutrophils, T cells, TLRs, and inflammatory cytokines. SARS-CoV-2 induces infection in the lung to activate an immune response in the gastrointestinal tract and disruption of epithelial cells in the lung (Fanos et al., 2020). SARS-CoV-2 induces alterations of lung microbiota such as a higher abundance of *Klebsiella oxytoca*, and *Rothia mucilaginosa* that drive inflammation in the lung (Han et al., 2020). Higher level of proinflammatory cytokines in the blood due to viral infections induce dysbiosis of gut bacteria and disruption of the gut barrier. Dysbiosis of gut microbiota breaks the integrity of gut barrier which induces the translocation of SARS-CoV-2 from the lung into the gut through the circulatory and lymphatic systems. Inflammation in the gut induces leaky gut that drives the development of sepsis in COVID-19 patients through translocation of bacterial antigens and toxins into the systemic circulation (**Figure 1**). Dysbiosis of gut microbiota also induces pathogenesis in the lung of respiratory distress syndrome (Aktas and Aslim, 2020). SARS-CoV-2 induces alterations of lung microbiota such as a higher abundance of *Klebsiella oxytoca*, and *Rothia mucilaginosa* that drive inflammation in the lung (Han et al., 2020).

*Streptococcus* sp.*, Escherichia* sp., and *Shigella* sp., showed a higher abundance in COVID-19 and H1N1 patients (Gu et al., 2020). The relative abundance of phyla *Actinobacteria and Firmicutes* was significantly reduced in H1N1 patients as compared to COVID-19 patients and healthy controls. At the family level, the number of Lachnospiraceae and Ruminococcaceae, which includes butyrate-producing bacteria (BPB) was drastically reduced in H1N1 patients. Further, the abundance of *Blautia, Agathobacter, Anaerostipes, Fusicatenibacter, Eubacterium hallii* group was significantly reduced. Recent findings also suggests that the frequency of *Fusicatenibacter, Anaerostipes, Agathobacter*, and *E. hallii* from the Lachnospiraceae family was significantly reduced in COVID-19 patients. On the one hand *Streptococcus, Rothia, Veillonella, Erysipelatoclostridium, and Actinomyces* were highly abundant in COVID-19 patients, and on the other hand, bacterial genera such as *Blautia*, *Romboutsia, Faecalibacterium, Fusicatenibacter*, *Collinsella*, *Bifidobacterium*, *and Eubacterium hallii* were reportedly high among healthy subjects. Patients of H1N1 Influenza have higher abundance of *Enterococcus, Prevotella, Finegoldia*, and *Peptoniphilus. Prevotella, Ezakiella, Murdochiella, and Porphyromonas* showed a higher abundance in H1N1 patients as compared to COVID-19 patients suggesting that respiratory viral pathogenesis seems to be determined also by inflammatory gut microbiome. H1N1-enriched bacteria has shown a positive association with inflammatory cytokines IL-2, IL-4, and IL-6, the later being the predominant culprit behind the onset of cytokine storm in COVID-19 (Gu et al., 2020). Whilst *Rothia* is involved in the development of pneumonia in immunocompromised individuals (Ramanan et al., 2014), both *Streptococcus and Rothia* are associated with disease severity in avian H7N9 virus infection (Lu et al., 2017). Further, H1N1 as well as H7N9 patients showed a higher abundance in opportunistic pathogens such as *Prevotella, Finegoldia, and Peptoniphilus* and elevated levels of IL-2 and IL-4 (Qin et al., 2015). The abundance of *Faecalibacterium prausnitzii, Eubacterium rectale, and Ruminococcus bromii* belong to Firmicutes and *Bifidobacterium adolescentis, Bifidobacterium pseudocatenulatum, and Collinsella aerofaciens* belong to Actinobacteria is reduced in COVID-19 patients. *B. adolescentis* prevents the activation of proinflammatory cytokine by inhibiting the activity of NF- κB (Yeoh et al., 2021). Dysbiosis of the lung microbiome enhances susceptibility to viral infections and also induces the development of secondary bacterial infections to increase mortality rates in COVID-19 patients. *Streptococcus salivarius* K12 showed inhibitory activity against SARS-CoV-2 (Di Pierro, 2020), and the abundance of certain oropharyngeal microbiomes such as haemophilus or streptococcus could likely contribute to severity of SARS-CoV-2 disease (https://doi.org/10.21203/rs.3.rs-127621/v1).

Given the aforementioned observations, it could be assumed that microbial dysbiosis in the lungs of COVID-19 patients likely predict the onset of hyperinflammation-driven ARDS. It has been shown that gut-associated *Enterobacteriaceae* showed a higher abundance in individuals progressing to develop ARDS. A higher abundance of Lachnospiraceae in ARDS patients showed an association with reduced survival. It is also hypothesised that microbiome alterations in gut and lung may predict the development of ARDS in COVID-19 patients (Dickson et al., 2020). Higher abundance of Proteobacteria and Bacteroidetes and a lower abundance of Firmicutes have been reported in influenza virus infection (Groves et al., 2018). Most prevalent bacterial genera such as *Acinetobacter, Chryseobacterium, Burkholderia, Brevundimonas, Sphingobium and Enterobacteriaceae* were reported in necropsy lung tissues of deceased patients with COVID-19. Among Acinetobacter, *Acinetobacter calcoaceticus, Acinetobacter baumannii, Acinetobacter nosocomialis, and Acinetobacter pittii*, with *A.baumannii* (AB) were highly abundant. Of the members belonging to Enterobacteriaceae, *Klebsiella, Escherichia coli, Proteus, and Enterobacte*r were highly abundant (Fan et al., 2020). Further, a higher abundance of *Clostridium hathewayi*, *Actinomyces viscosus*, and *Bacteroides nordii* has also been reported in antibiotic naïve COVID-19 patients. Antibiotic treated COVID-19 patients showed a significant depletion of beneficial symbionts such as *Fecalibacterium prausnitzii, Lachnospiraceae bacterium 5_1_63FAA, Eubacterium rectale, Ruminococcus obeum, and Dorea formicigenerans* in the gut relative to antibiotic naïve COVID-19 patients. Bacterial genus *Coprobacillus, Clostridium ramosum* and *Clostridium hathewayi* belong to phylum Firmicutes showed a positive correlation with COVID-19 severity whereas *Alistipes onderdonkii* and *F. prausnitzii* showed a negative association with disease severity. Furthermore, opportunistic pathogens such as *C.hathewayi, Bacteroides nordii, Actinomyces viscosus* appear to be highly abundant in the gut of COVID-19 patients. Bacteroidetes species such as *Alistipes onderdonkii* and *Bacteroides ovatus* showed a negative correlation with severity of COVID-19. *Bacteroides dorei, Bacteroides thetaiotaomicron, Bacteroides massiliensis*, and *Bacteroides ovatus* belonging to phylum Bacteroidetes showed an inverse negative correlation with SARS-CoV-2 viral loads in fecal samples of COVID-19 patients (Zuo et al., 2020). Interestingly, *B.dorei* has been shown to down-regulate ACE2 expression in the human colon (Yoshida et al., 2018). It also appears that *Alistipes* species are involved in tryptophan metabolism whereas *F. prausnitzii* showed anti-inflammatory properties (Vatanen et al., 2016).

Adding further the likely role of gut microbiota with disease progression, the outer membrane vesicles (OMVs) of *Acinetobacter nosocomialis* have been shown to augment inflammatory responses in epithelial cells (Nho et al., 2015). Enterobacteriaceae such as *E.coli, Klebsiella* sp., and *Proteus* sp. are colonisers of the gut albeit at low levels. It appears that the level of Enterobacteriaceae become elevated during inflammatory conditions engendering from exaggerated release of ROS and nitrogen intermediates by epithelial cells and transmigrating neutrophils in the gut lumen. Enterobactin of *E. coli* prevents the intracellular killing of action of myeloperoxidase of PMNs in the inflamed gut (Zeng et al., 2017). Gut *K. pneumoniae* enhances inflammatory response in human airway epithelial cells through activation of TLR4 and TLR2. Evidence also suggests that MAPKs p38, ERK and JNK regulates the secretion of inflammatory mediators and defensins from epithelial cells during Klebsiella infection. Klebsiella enhances inflammatory response by preventing the action of host proteins such as CYLD and MKP-1 which are involved in immune homeostasis post-inflammation. Klebsiella blocks IL-23/IL-17 and IL-12/IFN-γ signalling (Bengoechea and Sa Pessoa, 2019). Peptidoglycan and teichoic acid of streptococci bind to TLR-2 of epithelial and endothelial cells, monocytes and macrophages, which induce secretion of IL-1β, IL-6, IL-8, and TNF-α (Loughran et al., 2019). Actinomyces induces inflammatory lesions in tissues having PMNs, macrophages, and plasma cells (Engel et al., 1976). Besides, alteration in the gut microbiota has been demonstrated to have an association with several other respiratory infections, inflammatory bowel disease, depression, T2D, cardiovascular disease and hypertension (Schmidt et al., 2018). Studies have revealed that gut microbiota plays an eminent role in the pathogenesis of sepsis and ARDS (Dickson et al., 2016). Similarly, a study from China showed that some patients with COVID-19 presented with gut dysbiosis with decreased levels of *Lactobacillus* and *Bifidobacterium* (Xu et al., 2020).

Higher abundance of *Coprobacillus, Clostridium ramosum*, and *Clostridium hathewayi* led to disease severity in COVID-19 patients. Drugs such as chloroquine phosphate, lopinavir, ritonavir, and remdesivir used for the treatment of COVID-19 are also involved in gut dysbiosis. Application of short-term antibiotics use in COVID-19 treatment may also lead to alterations in gut bacteria. Prolonged use of doxycycline and hydroxychloroquine significantly reduced Bacteroidetes, Firmicutes, and Lactobacillus in the gut of COVID-19 patients. Interestingly, dysbiosis of gut bacteria is noticed even after recovery from COVID-19. FMT may be used to alter the gut microbiota in COVID-19 patients. It is essential to screen the presence of SARS-CoV-2 and gut microbiota profile in stool samples of patients after 35 days from recovery to provide the highest protection for FMT recipients. Stool samples of donors are also needed to screen the presence of SARS-CoV-2 (Kaźmierczak-Siedlecka et al., 2020).

## Gut Microbiome in the Elderly Covidants: Does It Explain High Mortality Rates?

The increased rates of mortality among the elderly in COVID-19 seem to stem from alterations in gut microbiota. The potential rationale underlying increased rates of mortality among the elderly in COVID-19 due to likely higher abundance of inflammatory bacteria (**Table 1**). The abundance of beneficial bifidobacterial may be depleted in the elderly peoples (Nagpal et al., 2018). Elderly people may be more susceptible to SARS-CoV-2 infection due to less diverse beneficial microorganisms.

**Table 1:** List of elevated bacteria in COVID-19 patients associated with inflammation and immunity.

Bacterial Genus/Species	Phylum	Mode of action associated with inflammation
*Streptococcus*	Firmicutes	Induces secretion of pro-inflammatory cytokines such as IL-1β, IL-6, IL-8, and TNF-α from epithelial cells.
*Actinomyces viscosus*	Actinobacteria	Induces inflammatory lesions in tissues having PMNs, macrophages, and plasma cells.
*Burkholderia*	Proteobacteria	Type VI effector, TecA of *B. cenocepacia* induces the activation of pyrin inflammasome through the deamidation of Rho GTPases that drive inflammation
*Klebsiella*		*K. pneumoniae* enhances inflammatory response in human airway epithelial cells through activation of TLR4 and TLR2 and preventing the action of host proteins such as CYLD and MKP-1 which are involved in immune homeostasis post inflammation event
*Escherichia coli*		Enterobactin of *E. coli* prevents action of bacteriocidal enzyme myeloperoxidase which is secreted from the neutrophil in the inflamed gut
*Acinetobacter baumannii*		HisF gene of this bacteria is responsible for the reduction of innate immune response.
*Acinetobacter nosocomialis*		Outer membrane vesicles (OMVs) induce inflammatory responses in epithelial cells

It has also been hypothesized that drugs used to treat diabetes mellitus and hypertension might upregulate the expression of ACE2 facilitating SARS-CoV-2 infection (Fang et al., 2020). Taking these factors into consideration, it can be easily speculated that SARS-CoV-2 infection might contribute to gut dysbiosis resulting in generalized inflammation contributing to MODS and other serious clinical worsening, especially in the elderly and patients with underlying clinical conditions.

Previous studies showed that dietary supplementation of probiotic formula with *Bifidobacterium lactis* in aged individuals enhanced the tumoricidal functions of natural killer (NK) cells. Probiotics such as *L. johnsonii, L. fermentum, L. reuteri, L. paracasei, L. rhamnosus, L. acidophilus, L. plantarum, belonged to genera Lactobacillus and B. longum, B. breve, B. bifidum, and B. animalis subsp. lactis* were involved in alleviating inflammatory manifestations *via* regulation of innate immune responses (Dhar and Mohanty, 2020). Probiotic bacteria like *L. rhamnosus, B. lactis, and B.breve* are involved in the down-regulation of inflammation through elevation of Treg cells (Feleszko et al., 2007).

Prebiotics such as inulin, fructo-oligosachharides (Fos), galactosachharides (Gos), and polydextrose are involved in the development of host immunity through alterations of gut microbiome. Prebiotics reportedly reduce the levels of the proinflammatory IL-6 that tends to be the prime culprit behind the hitherto described grave prognosis in COVID-19 and enhance the levels of anti-inflammatory IL-10 (West et al., 2017). Protein enriched diet enhances the abundance of gut commensals such as bifidobacteria and lactobacilli simultaneously reduces the pathogenic gut microbiota (Świątecka et al., 2011). Probiotic strains such as bifidobacteria or lactobacilli are not only involved in the clearance of virus from the respiratory tract but also augments the activity of antigen presenting cells, NK cells, T cells to drive the enhanced release of mucosal antibodies in lung fluids (Zelaya et al., 2016). *Lactobacillus casei* induces the phagocytic activity of alveolar macrophages and over expression of IgA, IFN-γ, and TNF-α in the host to protect against flu virus infections. *Bifidobacterium, Lactobacillus paracasei*, and *Lactobacillus rhamnosus* enhanced the efficacy of vaccine response against respiratory infections such as H1N1, H5N1, and H3N2 (He L. H. et al., 2020). Probiotic strains are involved in the regulation of proinflammatory and anti-inflammatory cytokines that likely could ameliorate ARDS complications in COVID-19.

Elderly individuals with hypertension, obesity, and diabetes are more prone to develop severe symptoms due to COVID-19 infections because dysbiosis of the gut microbiome reduces the integrity of the gut barrier, which in turn allows other pathogens to bind the enterocytes. Disruption of the integrity of tight junctions in between enterocytes of the gut called “leaky gut” in COVID-19 patients is responsible for the development of diarrhea, and inflammation due to higher levels of IL-6 in plasma and fecal calprotectin. This also allows SARS-CoV-2 to enter into the blood stream and bind with ACE2 of other body parts. *F. prausnitzii* belonging to class Clostridia and family Ruminococcaceae is responsible for the synthesis of a short-chain fatty acid (SCFA) such as butyric acid in the gut (**Figure 1**). The abundance of this bacteria was reduced in COVID-19 patients. Butyric acid maintains the integrity of gut barrier and shows anti-inflammatory activity through inhibition of NF-κB activity, activation of G protein-coupled receptors such as GPR41 and GPR43, suppression of histone deacetylase activity, and activation of regulatory T cells (Treg) cells. Fecal microbiota transplantation (FMT), and enhancement of abundance of next-generation probiotics such as butyrate-producing gut bacteria through daily intake of dietary fiber may be used to prevent inflammation and severity in COVID-19 patients (Kim, 2021). Protein extracts of whey and pea enhanced the abundance of Bifidobacterium, and Lactobacillus whereas reduced the abundance of pathogenic bacteria *Bacteroides fragilis* and *Clostridium perfringens* (Świątecka et al., 2011). SCFAs mainly acetate, propionate, and butyrate which are produced by the gut microbiota through the metabolism of resistant starches and dietary fibers provide energy to gut epithelial cells, maintain the integrity of the gut barrier, and suppressed inflammation by blocking the action of LPS and prevention of proinflammatory cytokine productions (Corrêa-Oliveira et al., 2016). Acetate may provide protection against respiratory syncytial virus (RSV) in the lung through the activation of IFN-β *via* GPR43 and IFNAR (Antunes et al., 2019).

Azithromycin which is a commonly used antibiotic for COVID-19 treatment reduced Shannon diversity index of bacterial communities particularly the abundance of Bifidobacterium genus. Other drugs such as metformin, statins, and psychiatric drugs are also involved in the alteration of the gut microbiota as well as enhance the risk of viral infections. Combinatorial approaches of probiotics, prebiotics, and natural products are used to control the balance of gut bacteria. Probiotics suppressed diarrhea by blocking the TLR expression and controlling the humoral and cellular immune responses. Bacterial genera such as Lactobacillus and Bifidobacterium showed strong antiviral action against influenza virus type A. These probiotics suppressed the growth of candida, *E. coli*, pseudomonas, and staphylococci during antibiotic administration in COVID-19 patients. Prebiotics and probiotics inhibit viral replication and infection *via* production of interferon (IFN) by activating plasmacytoid DCs *via* TLR9. LPS of Gram-negative and peptidoglycans (PG) of Gram-positive bacteria interact with viral proteins (Kiousi et al., 2019; Donati Zeppa et al., 2020). Gut microbiota effects on ACE2 at the gut and lung in such a way that probiotics may control the severity of the disease. Following gut colonization, probiotics could contribute to development of immunity against viral infections. Probiotics strains such as *Lactobacillus rhamnosus GG* and *Bifidobacterium longum* are involved in compressing the infection of ICU patients. Bacteriocins which are produced by Lactobacilli and Bifidobacteria are effective against pathogenic bacteria and viruses. Probiotic *Lactobacillus* sp. augments gut immunity through the synthesis of antiviral agents such as mucins and mucus in the intestine. Probiotics control innate and adaptive antiviral immunity through an interaction with dendritic cells, monocytes/macrophages, and lymphocytes. Lactic acid bacteria induces the synthesis of cytokines or chemokines through binding with intestinal epithelial cells *via* toll-like receptors. This also drives the abundance of IgA producing cells of bronchus, mammary glands and intestine which in turn stimulates mucosal immune system. Probiotics stimulate the secretion of IgG and IL-10 from the activated T-cells. It is essential to use probiotics along with prebiotics for the treatment of COVID-19 individuals (Din et al., 2021). Bacteriocin compounds such as staphylococcin 188, enterocin AAR-74, erwiniocin NA4 showed antiviral activity against HIV, HSV, Coliphage, influenza virus, and H1N1 virus (Gohil et al., 2021).

Together, gut microbiome alterations may play a paramount role in determining the clinical outcome of clinical COVID-19 with underlying co-morbid conditions like T2D, cardiovascular disorders, obesity, etc. Research is warranted to manipulate the profile of gut microbiota in COVID-19 by employing combinatorial approaches such as use of prebiotics, probiotics and symbiotics. Prediction of gut microbiome alterations in SARS-CoV-2 infection may likely permit the development of effective therapeutic strategies. Novel and targeted interventions by manipulating gut microbiota indeed represents a promising therapeutic approach against COVID-19 immunopathogenesis and associated co-morbidities. The impact of SARS-CoV-2 on host innate immune responses associated with gut microbiome profiling is likely to contribute to development of key strategies for application and has seldom been attempted, especially in the context of symptomatic as well as asymptomatic COVID-19 disease.

## Data Availability Statement

The original contributions presented in the study are included in the article/supplementary material. Further inquiries can be directed to the corresponding author.

## Author Contributions

IC and ES led the writing of this opinion article. All authors contributed to the article and approved the submitted.

## Funding

ES is supported by the Department of Science and Technology-Science and Engineering Research Board, Government of India (Grant number CRG/2019/006096), the Indian Council of Medical Research, Government of India (No. 45/2/2020-DDI/BMS) and the Swedish Research Council (VR 2014-02836).

## Conflict of Interest

The authors declare that the research was conducted in the absence of any commercial or financial relationships that could be construed as a potential conflict of interest.
